# The association between indoor biofuel use for cooking and glucose metabolism in adults: A cross-sectional study in Tanzania

**DOI:** 10.1371/journal.pgph.0003816

**Published:** 2025-05-14

**Authors:** Happyness Kunzi, Mussa K. Nsanya, Belinda Kweka, Evangelista Malindisa, Ng`wamba Sitta Ngissa, Bazil Baltazar. Kavishe, Kidola Jeremiah, Mette Frahm Olsen, Rikke Krogh-Madsen, Suzanne Filteau, Henrik Friis, Daniel Faurholt-Jepsen, George PrayGod

**Affiliations:** 1 Mwanza Research Centre, National Institute for Medical Research, Mwanza, Tanzania; 2 Department of Infectious Diseases, Rigshospitalet, Copenhagen, Denmark; 3 Department of Nutrition, Exercise and Sports, University of Copenhagen, Frederiksberg, Denmark; 4 Centre for Physical Activity Research, Copenhagen University Hospital, Rigshospitalet, Copenhagen, Denmark; 5 Faculty of Epidemiology and Population Health, London School of Hygiene and Tropical Medicine, London, United Kingdom; 6 Department of Clinical Medicine, University of Copenhagen, Copenhagen, Denmark; 7 Muhimbili Medical Research Centre, National Institute for Medical Research, Dar es Salaam, Tanzania; PLOS: Public Library of Science, UNITED STATES OF AMERICA

## Abstract

The burden of type 2 diabetes is rapidly increasing in low- and middle-income countries (LMICs), but determinants are not well-characterized. Household air pollution (HAP) from indoor biofuel use for cooking has been associated with non-communicable diseases and could be contributing to the increasing burden of diabetes in LMICs, though data are limited. We assessed the association between indoor biofuel use for cooking and glucose metabolism in HIV-infected and HIV-uninfected Tanzanian adults. This cross-sectional analysis included Tanzanian adults with and without HIV, from whom we collected sociodemographic and non-communicable disease risk factor data. The main predictor variable was indoor biofuel use for cooking, established using self-reported cooking location (indoor or outdoor) and fuel type (electricity/gas or biomass fuel), and categorized as minimal or no exposure, moderate exposure, and high exposure. Blood glucose and insulin were measured during oral glucose tolerance tests, allowing computation of outcome variables including markers of β-cell dysfunction (homeostatic model assessment-β, insulinogenic index, oral disposition index), insulin resistance (HOMA-IR and Matsuda index), and pre-diabetes and diabetes status. Logistic regression was used to assess associations, adjusting for age, sex, physical activity, smoking, socioeconomic status, HIV status, and body mass index. Among 1,871 participants (mean age 40.6 ± 11.9 years; 59.8% female), those with moderate and high exposure to HAP had approximately two-fold higher odds of a lower insulinogenic index (adjusted odds ratio [aOR] = 2.13, 95% CI: 1.27–3.57 and aOR = 2.31, 95% CI: 1.39–3.83, respectively) compared to those with minimal or no exposure. HAP was not associated with other markers of β-cell function, insulin resistance, pre-diabetes, or diabetes. In conclusion, HAP is associated with increased risk of β-cell dysfunction among individuals using biofuel for indoor cooking. Longitudinal studies using objective HAP measurements are needed to confirm these findings.

## Background

Diabetes is one of the leading non-communicable diseases (NCDs) globally. In 2019, 529 million people had diabetes and it is projected that 1.3 billion people will have this disease in 2050 [1]. Diabetes is increasing more rapidly and the proportion of mortality attributable to diabetes is higher in in low-and middle-income countries (LMICs) than in the high-income countries [2]. Type 2 diabetes risk may be elevated due to increased traditional risk factors such as physical inactivity, unhealthy diet, harmful use of alcohol and tobacco [3]. However, these potential risk factors cannot explain the excess burden of diabetes in LMICs including in Sub-Saharan Africa (SSA) [4]. Air pollution from ambient (outside sources like industrial and vehicle emission) but not household sources has been positively linked with insulin resistance and type 2 diabetes in high income countries (HICs) [5,6]. Accumulating evidence has highlighted the growing concern about HAP in LMICs and its role in type 2 diabetes development. Cross sectional studies in Thailand and China reported significant associations between exposure to HAP and type 2 diabetes [7,8]. A cohort study in China among middle and older adults without diabetes at baseline followed for 5 years found an increased risk of type 2 diabetes with exposure to HAP [9]. However, data are scarce in Africa, where available studies are small and have inconsistent results [10,11]. In SSA, few studies have linked ambient and HAP to the increased burden of cardiovascular and respiratory diseases but not with the other NCDs like type 2 diabetes [12,13].

HAP is generated when biomass fuels are burnt in poorly ventilated houses [14] resulting in smoke accumulation that contains particulate matter with low diameter (PM_2.5_) and harmful fumes like gaseous pollutants which contain carbon monoxide and formaldehyde [15–18]. Indoor smoke from biomas fuel can have 100 times higher levels of harmful fine particles than is acceptable and exposure to these fumes and PM_2.5_ may increase the risk of NCDs [16]. Oxidative stress is an important factor in the pathogenenesis of type 2 diabetes [19]. PM_2.5_ from biomass fuel when inhaled, may lead to overexpression of free radicals relative to antioxidants, with the imbalance resulting in oxidative stress [20]. Oxidative stress induces systemic inflammation which can damage body tissues like pancreas, liver and muscles leading to disruption of their functions; these could result in poor insulin secretion, insulin resistance and ultimately frank type 2 diabetes [21,22]. In addition, experimental studies have shown that when particulate matter (PM) deposits on the epithelial cells lining the airways, it activates inflammatory signaling pathways leading to inflammation [23]. Inflammation may damage the endothelium surface leading to reduced blood flow and impaired nutrients delivery to the pancreas which may negatively affect β-cell function [24] and compromise insulin secretion. It may also disrupt insulin signaling in skeletal muscles potentially increasing insulin resistance and elevating the risk of developing type 2 diabetes [21,25,26]. In Sub-Saharan Africa (SSA), approximately 90% of the population relies on biomass fuels as their primary source of energy for cooking. [27] In Tanzania, this figure is consistent, with petroleum and electricity contributing only 8% and 1.2%, respectively [28]. In Mwanza city, 96.5% of households rely on biofuels for cooking, indicating a high burden of household air pollution (HAP) [29]. There is a growing prevalence of type 2 diabetes in Tanzania [30] and while HAP may be contributing to the increased burden of type 2 diabetes in the region, data on this relationship remain limited [31,32]. We aimed to determine the association of HAP from biofuel cooking with β-cell dysfunction, insulin resistance and risk of pre-diabetes and diabetes among HIV infected and HIV uninfected Tanzanian adults.

## Methods

### Study design and setting

This was a cross-sectional analysis of baseline data from the Chronic Infections, Co-morbidities and Diabetes in Africa (CICADA) study, a cohort study investigating risk factors for diabetes among HIV-uninfected and HIV-infected adults in north-western Tanzania [33] and registered at clinical.trials.gov as NCT03106480. From October 2016 to November 2017, CICADA recruited 1947 participants and from these, participants with HAP, glucose and insulin data were eligible for inclusion in the current analysis.

### Sample size

We hypothesized that HAP would double the risk of either insulin resistance or β-cell dysfunction [21]. Assuming the prevalence of insulin resistance or β-cell dysfunction among participants without (or with minimal) exposure to HAP was 12% [33] and the ratio of non-exposed to HAP-exposed participants of was 0.15, we would need a minimum of 109 non-exposed and 719 HAP-exposed to demonstrate the association with odds ratio of 2, with 80% power at 5% significant level [34].

### Data collection

Data collection details for CICADA study have been described elsewhere [33]. Briefly, we collected data on sociodemographic variables (religion, education, occupation, income, and property ownership), levels of physical activity, fruits and vegetable intake as well as smoking and alcohol intake using a standardized questionnaire adapted from the World Health Organization (WHO) STEP wise approach to NCD risk factors Surveillance (STEPS) tool [35]. We collected data on antiretroviral therapy (ART) from HIV-infected participants and verified the information through participants’ ART cards. To classify exposure to indoor biofuel use for cooking that we named HAP [36], participants were asked if they were using biomass fuel (i.e., firewood, charcoal, crop residuals or animal dung), gas or electricity for cooking. We asked those using biomass fuel to provide information if the cooking was done indoors (i.e., in sleeping room, sitting room, corridor, or indoor kitchen) or in an outdoor kitchen. Since using gas or electricity for cooking produces less PM_2.5_ [37], such usage was categorized as having no or minimal exposure to HAP, using biomass fuel for cooking in an outdoor environment was categorized as having moderate exposure to HAP, and using biomass fuel for cooking indoors was categorized as having high exposure to HAP (14).

### Anthropometric measurements

Anthropometric measurements were done using standardized methods. With participants in minimal clothing, body weight and height measurements were taken in triplicate by well-trained personnel and median values were used for analyses. Body mass index (BMI) was computed as weight (kg)/height(m)^2^ and classified as underweight (BMI < 18.5 kg/m^2^), normal weight (BMI 18.5 to 24.9 kg/m^2^) and overweight or obese (BMI ≥ 25 kg/m^2^) as recommended by WHO [33].

### Assessment of outcome variables

Pre-diabetes and diabetes, β-cell dysfunction and insulin resistance were the main dependent variables. To assess these outcomes, participants were contacted one day prior to the clinic visit and instructed to come fasting for at least 8 hours. On arrival in the clinic, they provided venous blood for fasting blood glucose measurement (Hemocue 201 RT, Angelholm, Sweden) and fasting insulin which was later assessed using ELISA technique with dual-monoclonal antibodies (Salem, ALPCO, NH,) at the University of Copenhagen, Denmark. After fasting blood draw, participants were given 82.5 g of dextrose monohydrate (equivalent to 75 g of anhydrous glucose) diluted in 250 ml of drinking water to drink within 5 minutes for oral glucose tolerance test (OGTT), and blood specimens for glucose and insulin were collected after 30 minutes and 2 hours. According to WHO guidelines, 2-hour OGTT glucose level < 7.8 mmol/L was classified as normal and glucose level ≥ 7.8 to < 11.1 mmol/L was classified as impaired glucose tolerance (IGT), in this study termed as prediabetes (PD), and glucose level ≥ 11.1 mmol/L was classified as diabetes [38,39]

### Blood analyses

Venous blood from baseline and 30 and 120 minutes in the OGTT were separated and plasma aliquots for insulin analysis were stored at 80°C pending analysis. ELISA technique was used to assess insulin in Denmark using dual-monoclonal antibodies (ALPCO, Salem, NH, USA). HIV testing was done using two rapid antibody tests, i.e., SD Bioline HIV- 1/2 3.0 (Standard diagnostics Inc, South Korea),with positive tests confirmed using the Uni-Gold test (Trinity Biotech, Ireland). Discordant samples were tested using ELISA HIV bio kit (II Vironostika-HIV Ag/Ab Micro Elisa systems) (Biomerieux bv, The Netherlands).

We computed homeostatic model assessment-β (HOMA-β), insulinogenic index (IGI) and oral disposition index (ODI) as markers of β-cell function, and HOMA-insulin resistance (HOMA-IR) and Matsuda index as markers of insulin resistance [40,41] (S1 Table). Using Liu’s method and as described previously [42], these markers were dichotomized using optimal cut-off-points to indicate status of β-cell dysfunction and insulin resistance. β-cell dysfunction was defined as HOMA-β index <38.3 (mU/L)/(mmol/L), IGI < 0.71 (mU/L)/(mmol/L), ODI < 0.16 (mU/L) (mgdL)(mU/L)^-1^. Insulin resistance was HOMA-IR index >1.9 (mU/L)/(mmol/L) or Matsuda index <7.2 (mU/L)/(mmol/L).

### Ethical consideration

CICADA study received ethical approval from the Medical Research Coordinating Committee of the National Institute for Medical Research in Tanzania, the ethics committee of the London School of Hygiene and Tropical Medicine in UK and a consultative approval from the National Committee on Health Research Ethics in Denmark. All participants were fully informed of the purpose, procedures, benefits, risks and their rights while in the study. Written informed consent was sought from all eligible participants prior to enrolment. Those who were diagnosed with pre-diabetes were counselled on diet and lifestyle and those with diabetes were referred to a nearby public health facility for further management.

### Statistical analysis

Data were analyzed using Stata version 16 (STATA Corp LLC, College Station, Texas, USA). Categorical variables were presented as counts with percentages and their differences assessed using chi-squared test. Socioeconomic status was computed using principal component analysis and the first component divided into terciles to establish levels of socio-economic status for analysis. Differences in median levels of markers of beta-cell dysfunction and insulin resistance in those with minimal HAP exposure and higher HAP exposures were compared using Wilcoxon rank-sum test. To investigate the association between HAP with β-cell dysfunction and insulin resistance, logistic regression was used while the association between HAP and pre-diabetes and diabetes was assessed using multinomial logistic regression. Directed acyclic graph (DAG) was constructed based on existing literature and theoretical understanding on links between HAP and study outcomes to guide the selection of confounders [43]. Based on this framework, age, sex, smoking, physical activity, socioeconomic status, HIV status and BMI were adjusted for as confounders in final models. We tested if HIV and BMI categories modified the association between exposure and outcomes in both logistic and multinomial logistic regression. Results were presented as odds ratio (OR) and relative risk ratio (RRR) with corresponding 95% confidence interval (CI). *P* value of <0.05 indicated significant differences.

## Results

Of 1947 participants from the CICADA cohort, 1871 (96.1%) participants were analysed, the mean (±SD) age was 40.6 (±11.9) years and 1119 (59.8%) were females. Most participants had primary education and were self-employed in small businesses. (**Table 1**). Neither HIV nor BMI status modified the association between HAP and pre-diabetes and diabetes and other outcomes.

**Table 1:** Background characteristics of participants by sex.

Participants Characteristics	Categories	Women n(%)	Men n (%)
Age groups (years)	18-30	289 (25.8)	118 (15.7)
	31-40	375 (33.5)	223 (29.7)
	41-50	282 (25.2)	216 (28.7)
	>50	173 (15.5)	195 (25.9)
Educational level	No formal education	217 (19.4)	90 (12.0)
	Primary education	755 (67.5)	523 (69.5)
	Secondary/tertiary college	147 (13.1)	139 (18.5)
Socioeconomic status	Lower tertile	365 (32.6)	250 (33.2)
	Middle tertile	369 (33.0)	254 (33.8)
	Upper tertile	385 (34.4)	248 (33.0)
HIV status	Negative	362 (32.3)	265 (35.2)
	Positive not antiretroviral therapy	561 (50.1)	363 (48.3)
	Positive on antiretroviral therapy	196 (17.5)	124 (16.5)
Body mass index	Underweight(kg/m^2^)	199 (17.8)	210 (27.9)
	Normal weight(kg/m^2^)	617 (55.1)	467 (62.1)
	Overweight or obese(kg/m^2^)	303 (27.1)	75 (10.0)
Fruits and vegetable consumption (servings)	0	118 (10.6)	111 (14.9)
	1-2	601 (54.1)	380 (50.9)
	3-4	238 (21.4)	163 (21.8)
	≥4	154 (13.9)	93 (12.4)
Physical activity	Not active (<600 MET minutes per week)	80 (7.1)	115 (15.3)
	Active (≥600 MET minutes per week))	1,039 (92.9)	637 (84.7)
Smoking	Never	1059 (94.5)	364 (48.5)
	Ex-smoker	50 (4.4)	222 (29.6)
	Current smoker	12 (1.1)	164 (21.87)
Alcohol consumption	Never	390 (34.8)	142 (18.9)
	Ex- drinker	468 (41.7)	293 (39.1)
	Current- drinker	263 (23.5)	315 (42,0)
Household air pollution	Minimal or no exposure	43 (3.8)	70 (9.3)
	Moderate exposure	516 (46.1)	306 (40.7)
	High exposure	560 (50.1)	376 (50.0)

MET, Metabolic equivalent of tasks.

We found that compared to minimal or no-exposure group, participants in either moderately or highly HAP-exposed groups had significant lower median insulinogenic index (0.98 vs 1.34, *p* = 0.01) and (1.04 vs 1.34, *p* = 0.01), respectively) (**Table 2**). Although in general other markers of β-cell dysfunction were lower in both moderately-exposed and highly-exposed groups compared to minimally-exposed groups, these differences did not reach statistical significance (**Table 2**). There were no differences in median HOMA-IR, Matsuda index or glucose comparing minimal or no-exposure group with moderately or highly HAP-exposed groups.

**Table 2:** Distribution of glucose metabolic markers according to levels of household air pollution.

Markers of glucose metabolism	Minimal/no exposure for HAP	Moderate exposure for HAP	High exposure for HAP	P^1^	P^2^
HOMA-β index (mU/L, mmol/L), median (IQR)	38.74 (21.54, 60.48)	35.98 (21.19,56.16)	35.62 (22.59, 56.91)	0.19	0.34
Oral disposition index (mU/L)/(mg/dL)(mU/ L)-1, median (IQR)	0.22 (0.14,0.37)	0.20 (0.11, 0.35)	0.20 (0.11,0.35)	0.12	0.13
Insulinogenic index(mU/L/mg/dL), median (IQR)	1.34 (0.80,2.01)	0.98 (0.51,1.75)	1.04 (0.52, 1.94)	0.01	0.01
HOMA-IR(mU/L, mmol/L), median (IQR)	1.62 (0.91,2.57)	1.54 (0.91, 2.35)	1.50 (0.96, 2.47)	0.28	0.40
Matsuda index(mU/L, mg/dL), median (IQR)	5.64 (4.11, 9.73)	6.42 (4.47, 9.69)	6.28 (4.29, 9.41)	0.16	0.40
Two hours oral glucose tolerance n(%) median (IQR)	7.6 (6.9, 8.9)	7.7 (0.7, 8.8)	7.8 (7.1, 8.9)	0.96	0.75

1 Difference between minimal and moderate exposure.

2 Difference between minimal and upper exposure.

HAP, indoor air pollution.

In fully adjusted model, adjusted for age, sex, smoking physical activity, HIV status, BMI and socioeconomic status we found that moderate and high exposure to HAP were associated with higher odds of lower insulinogenic index ((aOR 2.13, 95% CI:1.27, 3.57) and (aOR 2.31, 95% CI: 1.39, 3.83)), respectively (Table 3). Also, in fully adjusted model, moderate exposure and high exposure to HAP were associated with higher odds of lower oral disposition index ((aOR 1.52, 95% CI:0.97, 2.37) and ((aOR 1.47, 95% CI:0.95, 2.28)), respectively. However, these estimates did not reach statistical significance (Table 3).

**Table 3:** Association of household air pollution with β-cell dysfunction among adults living in Mwanza, Tanzania.

		Reduced HOMA-β^1^				Reduced oral disposition index^2^				Reduced insulinogenic index^3^			
Household air pollution	n (%)	OR (95% CI)4	P	aOR (95% CI)5	P	OR (95% CI)4	P	aOR (95% CI)5	P	OR (95% CI)4	P	aOR (95% CI)5	P
Minimal or no exposure	113 (6.04)	Reference		Reference		Reference		Reference		Reference		Reference	
Moderate exposure	822 (4.93)	1.40 (0.90, 2.05)	0.15	0.99 (0.62,1.58)	1.00	1.44 (0.93, 2.21)	0.09	1.52 (0.97, 2.37)	0.06	2.41 (1.47, 3.96)	0.01	2.13 (1.27, 3. 57)	0.01
High exposure	936 (50.03)	1.26 (0.84,1.90)	0.27	1.16 (0.73,1.83)	0.52	1.42 (0.93, 2.18)	0.11	1.47 (0.95, 2.28)	0.08	2.33 (1.42, 3.82)	0.01	2.31 (1.39,3.83)	0.01

HOMA- β- Homeostatic model assessment-β.

aOR, Odds ratio ^1^ < 38.3(mU/L)(mmol/L) ^2^ < 0.16(mU/L)(mg/dL)(mU/L) ^3^ < 0.7(mU/L)mg/d ^4^Model adjusted for age and sex, ^5^Model adjusted for age, sex, smoking, physical activities, socioeconomic status, HIV status, e and body mass index.

There were no associations of HAP with HOMA-β in adjusted analysis. We found no association between HAP and insulin resistance markers and pre-diabetes and diabetes in adjusted models (Tables 4 and 5).

**Table 4:** Association of household air pollution with insulin resistance.

		Increased HOMA-IR^1^				Reduced Matsuda index^2^			
Household air pollution	n(%)	OR (95% CI)3	P	aOR (95% CI)4	P	OR (95% CI)3	P	aOR (95% CI)4	P
Minimal or no exposure	113 (6.04)	Reference		Reference		Reference		Reference	
Moderate exposure	822 (43.93)	0.58 (0.38, 0.87)	0.01	0.82 (0.51, 1.31)	0.50	0.89 (0.59,1.35)	0.58	1.33 (0.83, 2.12)	0.24
High exposure	936 (50.3)	0.66 (0.43,1.00)	0.04	0.75 (0.47,1.18)	0.21	0.91 (0.60,1.37)	0.66	1.05 (0.67; 1.66)	0.82

HOMA-IR- Homeostatic model assessment-Insulin Resistance.

OR, Odds Ratio ^1^ > 1.9(mU/L)(mmol/L) ^2^ < 7.2(mU/L)(mg/dL) ^3^Model adjusted for age and sex ^4^Models adjusted for age, sex, smoking, physical activities, socioeconomic status, HIV status, and body mass index.

**Table 5:** Association of household air pollution with Pre-diabetes and diabetes among adults living in Mwanza, Tanzania.

		Minimally-adjusted model^1^				Fully-adjusted model^2^			
Household air pollution	n (%)	Pre-diabetes aRRR (95% CI)	P	Diabetes aRRR (95% CI)	P	Pre-diabetes aRRR (95% CI)	P	Diabetes aRRR (95% CI)	P
Minimal or no exposure	113 (6.04)	Reference		Reference		Reference		Reference	
Moderate exposure	822 (43.93)	1.02 (0.67, 1.54)	0.94	1.10 (0.47, 2.57)	0.83	1.00 (0.66, 1.53)	0.99	1.05 (0.44, 2.55)	0.90
High exposure	936 (50.03)	1.11 (0.74, 1.68)	0.61	1.02 (0.44, 2.38)	0.96	1.13 (0.74, 1.72)	0.57	0.99 (0.41, 2.40)	0.99

aRRR-Relative Risk Ratio ^1^Model adjusted for age and sex. ^2^Model adjusted for age, sex, physical activity, smoking, HIV status and body mass index.

## Discussion

In this cross-sectional study we report that moderate and high exposure to HAP were associated with higher odds of β-cell dysfunction defined by IGI. There was no association between HAP with insulin resistance as defined by HOMA-IR and Matsuda index and we found no association between HAP and pre-diabetes and diabetes or other markers of β-cell function.

IGI measures the extent to which insulin is produced in response to glucose load [44], and low IGI indicates impaired first phase of insulin secretion which is a predictor of diabetes [36]. Air pollution can negatively affect glucose metabolism by interfering with pancreatic β-cell function hence decreasing insulin secretion [45]. Although cooking outdoors is considered to be less harmful than cooking indoors [46], our findings suggest that the use of biomass fuel both indoors and outdoors could lead to reduced β-cell function which potentially increases the risk of future diabetes [17]. Our results are similar to study of rural women in China which found that HAP was associated with poor insulin secretion, especially among women cooking for a long duration of time [47]. HAP may induce oxidative stress, trigger systemic inflammation and endothelia dysfunction, and lead to damage of body tissues including the pancreas resulting in reduced β-cell function and lower insulin secretion [21].

Poverty is a major reason for the use of biofuel for cooking [48]. Thus, most biomass users might not afford taking adequate fruits and vegetables which are good sources of antioxidants. Inadequate intake of fruits and vegetables may increase oxidative stress which could further contribute to elevating the risk of lower β-cell function.

Our study found no significant association between HAP and insulin resistance as defined by HOMA-IR and Matsuda index. Our findings are similar to a meta-analysis of the association of air pollution with insulin resistance, which found no significant association with low diameter particulate matter (PM_2.5_) but found significant association with larger diameter particulate matter [5]. Our results on insulin resistance are contrary to the KORA study, a longitudinal study conducted in Augsburg in Germany which investigated the association between air pollution and insulin sensitivity using HOMA-IR and found that long exposure to air pollution with PM_2.5_ was a risk factor for impaired insulin sensitivity [6]. However, this study unlike our study investigated ambient air pollution which is related to strong airborne pollutants from traffic and industrial emissions which may differ from air pollution found at home in terms of pollutant composition and concentration. Further studies are warranted to confirm relations of HAP and insulin resistance in LMICs.

Despite the finding that HAP was associated with a higher odd of β-cell dysfunction, HAP was not associated with prediabetes and diabetes. This could have been due at least two reasons. First, we assessed HAP using reported rather than objective measurements. This could have led to misclassification of exposures resulting in lack of association. Second, the reduced β-cell function due to HAP may not have been adequate to trigger development of overt diabetes given existing compensatory mechanisms in the body preventing dysglycaemia. In contrary to our study, an exploratory analysis in India using data collected through India`s third national family health survey which drew a sample of 124,385 participants found that the use of solid fuel for cooking increased diabetes risk [49].

Similar to the Indian study, another study among Honduras women in Central America where HAP was measured by collecting PM_2.5_ and checked using aircheck devices found participants who were HAP-exposed were at higher risk of having pre-diabetes or diabetes [17]. These Indian and Honduras studies suggest that exposure to HAP and existence of multiple risk factors might increase the risk of diabetes among people exposed to HAP. Future studies should explore these associations among groups with higher risk of HAP in order to provide evidence for developing interventions targeting these at-risk populations.

Our results, call for increased awareness of the health risks associated with biomass fuel use in SSA to help communities make informed decisions on use of biomass fuel. In addition, health programs should be designed to reduce indoor air pollution as a strategy for reducing diabetes burden. To achieve this, NCDs control programs should integrate education on improving cooking environment in diabetes prevention and care education modules. In addition, wider public health policies aimed at reducing air pollution should be encouraged as these will have the added benefit of protecting pancreatic function and reducing the burden of diabetes.

## Strengths and limitations

This study had a large sample size therefore had high precision around risk estimates and large power to detect associations or differences between groups. More than 96% of data from the origin cohort were analysed, therefore, selection bias due to missing data is likely to be very minimal. Moreover, the study was able to adjust for traditional risk factors hence reducing the risk of confounding and it included both HIV infected and non-infected individuals, thus it can be generalized. However, the study depended on self-reported, rather than measured data to assess HAP which might have led to recall bias and misclassification of exposure leading to reduced strength of association between HAP and study outcomes. In addition, detailed data on the exact types and quantities of biofuels used were not collected. The classification of exposure levels was based on the cooking location (indoor versus outdoor), which may have introduced inaccuracies in exposure categorization. Moreover, the use of biomass fuel is linked to poor social economic status as we reported [50], individuals in lower socioeconomic groups might underreport the extent of their poverty or financial struggles to avoid stigma or negative judgment. Conversely, those in higher socioeconomic status groups might exaggerate their wealth or success to fit societal expectations. Therefore, social desirability bias may have been introduced in this study [51] and may have led to over-reporting or under reporting of HAP exposure. This bias could affect the accuracy of the reported data and, consequently, the generalizability of the findings. Future studies using objective measure of HAP are warranted. Also, this was a cross-sectional study and causality cannot be confirmed as HAP might have led to lower IGI through oxidative stress, systemic inflammation and endothelium dysfunction mechanisms [52]. However, chronic diseases including diabetes are associated with increased medical expenses and low productivity that limits access to clean fuel and increased exposure to HAP among individuals with diabetes [53]. Future research employing longitudinal designs are needed to better establish causality and determine the direction of the relationship.

## Conclusion and recommendations

HAP may impair insulin β-cell function and could contribute to a higher burden of diabetes in SSA. Based on these findings, we recommend the inclusion of HAP in the policy toward reduction of diabetes risk and promotion of community awareness on the health impact due to the use of biofuel for cooking purposes. To better understand HAP and links to diabetes, future studies should use objective HAP assessment to determine the dosage and the exposure period of inhaling smoke. Also, to better understand the mechanism behind the association for HAP and impaired glucose metabolism, we recommend for studies measuring the inflammatory markers levels, oxidative stress indicators and insulin resistance and β-cell function markers in relation to HAP exposure.

## Supporting information

S1 Table Formulae for β-cell function and insulin resistance markers. (PDF)

## References

[1] Ong KL, Stafford LK, McLaughlin SA, Boyko EJ, Vollset SE, Smith AE, et al. Global, regional, and national burden of diabetes from 1990 to 2021, with projections of prevalence to 2050: a systematic analysis for the Global Burden of Disease Study 2021. The Lancet. 2023. 10.1016/S0140-6736(23)01301-6 PMC10364581 37356446

[2] World Health Organization. Global report on diabetes. Geneva: World Health Organisation; 2016 Contract No.: ISBN: 9789241565257.

[3] Milanzi EB, Nkoka O, Kanje V, Ntenda PAM. Air pollution and non-communicable diseases in sub-saharan africa. Sci Afr. 2021;11.

[4] Dagenais GR, Gerstein HC, Zhang X, McQueen M, Lear S, Lopez-Jaramillo P, et al. Variations in Diabetes Prevalence in Low-, Middle-, and High-Income Countries: Results From the Prospective Urban and Rural Epidemiological Study. Diabetes Care. 2016;39(5):780–7. doi: 10.2337/dc15-2338 26965719

[5] Dang J, Yang M, Zhang X, Ruan H, Qin G, Fu J, et al. Associations of Exposure to Air Pollution with Insulin Resistance: A Systematic Review and Meta-Analysis. Int J Environ Res Public Health. 2018;15(11):2593. doi: 10.3390/ijerph15112593 30463387 PMC6266153

[6] Zhang S, Mwiberi S, Pickford R, Breitner S, Huth C, Koenig W, et al. Longitudinal associations between ambient air pollution and insulin sensitivity: results from the KORA cohort study. Lancet Planet Health. 2021;5(1):e39–49. doi: 10.1016/S2542-5196(20)30275-8 33421408

[7] Juntarawijit C, Juntarawijit Y. Cooking with biomass fuel and cardiovascular disease: a cross-sectional study among rural villagers in Phitsanulok, Thailand. F1000Res. 2020;9:307. doi: 10.12688/f1000research.23457.2 33093943 PMC7549177

[8] Chen Y, Feng S, Chang Z, Zhao Y, Fu J, Liu Y. Household solid fuel use with diabetes and fasting blood glucose levels among middle-aged and older adults in China. Environ Sci Pollut Res. 2022;29(45):68247–56. 10.1007/s11356-022-20591-6 35538340

[9] Liu Y, Shao J, Liu Q, Zhou W, Huang R, Zhou J, et al. Association between household fuel combustion and diabetes among middle-aged and older adults in China: A cohort study. Ecotoxicol Environ Saf. 2023;258:114974. doi: 10.1016/j.ecoenv.2023.114974 37150109

[10] Brook RD, Xu X, Bard RL, Dvonch JT, Morishita M, Kaciroti N, et al. Reduced metabolic insulin sensitivity following sub-acute exposures to low levels of ambient fine particulate matter air pollution. Sci Total Environ. 2013;448:66–71. doi: 10.1016/j.scitotenv.2012.07.034 22901427 PMC4391076

[11] Thiering E, Cyrys J, Kratzsch J, Meisinger C, Hoffmann B, Berdel D, et al. Long-term exposure to traffic-related air pollution and insulin resistance in children: results from the GINIplus and LISAplus birth cohorts. Diabetologia. 2013;56(8):1696–704. doi: 10.1007/s00125-013-2925-x 23666166 PMC3699704

[12] Mocumbi AO, Stewart S, Patel S, Al-Delaimy WK. Cardiovascular Effects of Indoor Air Pollution from Solid Fuel: Relevance to Sub-Saharan Africa. Curr Environ Health Rep. 2019;6(3):116–26. doi: 10.1007/s40572-019-00234-8 31102183

[13] Glenn BE, Espira LM, Larson MC, Larson PS. Ambient air pollution and non-communicable respiratory illness in sub-Saharan Africa: a systematic review of the literature. Environ Health. 2022;21(1):40. doi: 10.1186/s12940-022-00852-0 35422005 PMC9009030

[14] Conant J, Fadem P. A community guide to environmental health. Hesperian Foundation; 2008.

[15] Bartington SE, Bakolis I, Devakumar D, Kurmi OP, Gulliver J, Chaube G, et al. Patterns of domestic exposure to carbon monoxide and particulate matter in households using biomass fuel in Janakpur, Nepal. Environ Pollut. 2017;220(Pt A):38–45. doi: 10.1016/j.envpol.2016.08.074 27707597 PMC5157800

[16] World Health Organization. Household air pollution internet. 2024 [updated 16 October 2024]. Available from: https://www.who.int/news-room/fact-sheets/detail/household-air-pollution-and-health

[17] Rajkumar S, Clark ML, Young BN, Benka-Coker ML, Bachand AM, Brook RD, et al. Exposure to household air pollution from biomass-burning cookstoves and HbA1c and diabetic status among Honduran women. Indoor Air. 2018;:10.1111/ina.12484. doi: 10.1111/ina.12484 29896912 PMC6292747

[18] Li L, Yang A, He X, Liu J, Ma Y, Niu J, et al. Indoor air pollution from solid fuels and hypertension: A systematic review and meta-analysis. Environ Pollut. 2020;259:113914. doi: 10.1016/j.envpol.2020.113914 31935611

[19] Rehman K, Akash MSH. Mechanism of Generation of Oxidative Stress and Pathophysiology of Type 2 Diabetes Mellitus: How Are They Interlinked?. J Cell Biochem. 2017;118(11):3577–85. doi: 10.1002/jcb.26097 28460155

[20] Valavanidis A, Fiotakis K, Vlachogianni T. Airborne particulate matter and human health: toxicological assessment and importance of size and composition of particles for oxidative damage and carcinogenic mechanisms. J Environ Sci Health C Environ Carcinog Ecotoxicol Rev. 2008;26(4):339–62. doi: 10.1080/10590500802494538 19034792

[21] Rajagopalan S, Brook RD. Air pollution and type 2 diabetes: mechanistic insights. Diabetes. 2012;61(12):3037–45. doi: 10.2337/db12-0190 23172950 PMC3501850

[22] Hectors TLM, Vanparys C, van der Ven K, Martens GA, Jorens PG, Van Gaal LF, et al. Environmental pollutants and type 2 diabetes: a review of mechanisms that can disrupt beta cell function. Diabetologia. 2011;54(6):1273–90. doi: 10.1007/s00125-011-2109-5 21442161

[23] Utell MJ, Frampton MW, Zareba W, Devlin RB, Cascio WE. Cardiovascular effects associated with air pollution: potential mechanisms and methods of testing. Inhal Toxicol. 2002;14(12):1231–47. doi: 10.1080/08958370290084881 12454788

[24] Li C, Fang D, Xu D, Wang B, Zhao S, Yan S. Mechanisms in endocrinology: main air pollutants and diabetes-associated mortality: a systematic review and meta-analysis. Eur J Endocrinol. 2014;171(5):R183–90. 10.1530/EJE-14-0287 25298377

[25] Sun Q, Yue P, Deiuliis JA, Lumeng CN, Kampfrath T, Mikolaj MB, et al. Ambient air pollution exaggerates adipose inflammation and insulin resistance in a mouse model of diet-induced obesity. Circulation. 2009;119(4):538–46. doi: 10.1161/CIRCULATIONAHA.108.799015 19153269 PMC3845676

[26] Liu C, Ying Z, Harkema J, Sun Q, Rajagopalan S. Epidemiological and experimental links between air pollution and type 2 diabetes. Toxicol Pathol. 2013;41(2):361–73. doi: 10.1177/0192623312464531 23104765 PMC3988529

[27] International Enargy Agency. Energy and Air Pollution: World Energy Outlook Special Report; 2016.

[28] Felix M, Gheewala SH. A Review of Biomass Energy Dependency in Tanzania. Energy Procedia. 2011;9:338–43. doi: 10.1016/j.egypro.2011.09.036

[29] PrayGod G, Mukerebe C, Magawa R, Jeremiah K, Török ME. Indoor Air Pollution and Delayed Measles Vaccination Increase the Risk of Severe Pneumonia in Children: Results from a Case-Control Study in Mwanza, Tanzania. PLoS One. 2016;11(8):e0160804. doi: 10.1371/journal.pone.0160804 27508389 PMC4979871

[30] Chillo O, Mzokolo I, Peter E, Malindisa E, Thabit H, Tungu A. Type 2 diabetes mellitus in Tanzania. A narrative review of epidemiology and disease trend. Curr Diabetes Rev. 2025;21(1):E030124225188. 10.2174/0115733998267513231208100124 38173215

[31] Oluwatosin D, Ola K, Olayinka A. The impacts of the use of biomass solid fuels for household cooking in sub-saharan africa-a review. Journal of Environmental Management. 2022;300:113800.

[32] Sulaiman C, Abdul-Rahim AS. The Impact of Wood Fuel Energy on Economic Growth in Sub-Saharan Africa: Dynamic Macro-Panel Approach. Sustainability. 2020;12(8):3280. doi: 10.3390/su12083280

[33] Jeremiah K, Filteau S, Faurholt-Jepsen D, Kitilya B, Kavishe BB, Krogh-Madsen R, et al. Diabetes prevalence by HbA1c and oral glucose tolerance test among HIV-infected and uninfected Tanzanian adults. PLoS One. 2020;15(4):e0230723. doi: 10.1371/journal.pone.0230723 32267855 PMC7141607

[34] Sullivan KM. Sample Size:X-Sectional, Cohort, & Randomized Clinical Trials Emory University: Emory University; 2013 [updated 2013/04/06; cited 2023 12/12]. Available from: https://www.openepi.com/SampleSize/SSCohort.htm

[35] World Health Organization. STEP wise approach to NCD risk factor surveillance (STEPS) Gevener, Switzerland. 2002 [cited 2023 06 May]. Noncommunicable Disease Surveillance, Monitoring and Reporting]. Available from: http://www.who.int/chp/steps/manual/en/

[36] Hanefeld M, Koehler C, Fuecker K, Henkel E, Schaper F, Temelkova-Kurktschiev T, et al. Insulin secretion and insulin sensitivity pattern is different in isolated impaired glucose tolerance and impaired fasting glucose: the risk factor in Impaired Glucose Tolerance for Atherosclerosis and Diabetes study. Diabetes Care. 2003;26(3):868–74. doi: 10.2337/diacare.26.3.868 12610051

[37] Puzzolo E, Pope D. Clean fuels for cooking in developing countries. ESST. 2017:289-97.

[38] World Health Organization. Definition and diagnosis of diabetes mellitus and intermediate hyperglycaemia [internet]. Geneva, Switzerland; 2006.

[39] Edelstein SL, Knowler WC, Bain RP, Andres R, Barrett-Connor EL, Dowse GK, et al. Predictors of progression from impaired glucose tolerance to NIDDM: an analysis of six prospective studies. Diabetes. 1997;46(4):701–10. doi: 10.2337/diab.46.4.701 9075814 PMC2517225

[40] Albareda M, Rodríguez-Espinosa J, Murugo M, de Leiva A, Corcoy R. Assessment of insulin sensitivity and beta-cell function from measurements in the fasting state and during an oral glucose tolerance test. Diabetologia. 2000;43(12):1507–11. doi: 10.1007/s001250051561 11151759

[41] Utzschneider KM, Prigeon RL, Faulenbach MV, Tong J, Carr DB, Boyko EJ, et al. Oral disposition index predicts the development of future diabetes above and beyond fasting and 2-h glucose levels. Diabetes Care. 2009;32(2):335–41. doi: 10.2337/dc08-1478 18957530 PMC2628704

[42] Liu X. Classification accuracy and cut point selection. Stat Med. 2012;31(23):2676–86. doi: 10.1002/sim.4509 22307964

[43] Laubach ZM, Murray EJ, Hoke KL, Safran RJ, Perng W. A biologist’s guide to model selection and causal inference. Proc Biol Sci. 2021;288(1943):20202815. doi: 10.1098/rspb.2020.2815 33499782 PMC7893255

[44] Potter KJ, Boudreau V, Bonhoure A, Tremblay F, Lavoie A, Carricart M, et al. Insulinogenic index and early phase insulin secretion predict increased risk of worsening glucose tolerance and of cystic fibrosis-related diabetes. J Cyst Fibros. 2023;22(1):50–8. doi: 10.1016/j.jcf.2022.07.014 36028423

[45] Toledo-Corral CM, Alderete TL, Habre R, Berhane K, Lurmann FW, Weigensberg MJ, et al. Effects of air pollution exposure on glucose metabolism in Los Angeles minority children. Pediatr Obes. 2018;13(1):54–62. doi: 10.1111/ijpo.12188 27923100 PMC5722706

[46] Rajper SA, Nazir A, Ullah S, Li Z. Perceived health risks of exposure to indoor air pollution from cooking fuels in Sindh, Pakistan. Pol J Environ Stud. 2020;29(4).

[47] Kang N, Song X, Zhang C, Li R, Yuchi Y, Liao W, et al. Association of household air pollution with glucose homeostasis markers in Chinese rural women: Effect modification of socioeconomic status. Ecotoxicol Environ Saf. 2022;248:114283. doi: 10.1016/j.ecoenv.2022.114283 36371884

[48] Muindi K, Egondi T, Kimani-Murage E, Rocklov J, Ng N. “We are used to this”: a qualitative assessment of the perceptions of and attitudes towards air pollution amongst slum residents in Nairobi. BMC Public Health. 2014;14:226. doi: 10.1186/1471-2458-14-226 24597487 PMC3973865

[49] Mishra S, McClure L, Golla V, Guddattu V, Lungu C, Sathiakumar N. Relationship between diabetes mellitus and indoor air pollution: an exploratory analysis. Int J Noncommun Dis. 2020;5(4):165–70.

[50] Zhou Z, Dionisio KL, Arku RE, Quaye A, Hughes AF, Vallarino J, et al. Household and community poverty, biomass use, and air pollution in Accra, Ghana. Proc Natl Acad Sci U S A. 2011;108(27):11028–33. doi: 10.1073/pnas.1019183108 21690396 PMC3131368

[51] Grimm P. Social desirability bias. 2010.

[52] Bonanni LJ, Wittkopp S, Long C, Aleman JO, Newman JD. A review of air pollution as a driver of cardiovascular disease risk across the diabetes spectrum. Front Endocrinol (Lausanne). 2024;15:1321323. doi: 10.3389/fendo.2024.1321323 38665261 PMC11043478

[53] Seuring T, Archangelidi O, Suhrcke M. The Economic Costs of Type 2 Diabetes: A Global Systematic Review. Pharmacoeconomics. 2015;33(8):811–31. doi: 10.1007/s40273-015-0268-9 25787932 PMC4519633

