# Supplementary material for: The association between indoor biofuel use for cooking and glucose metabolism in adults: A cross-sectional study in Tanzania
Source: PLOS Glob Public Health. 2025 May 14;5(5):e0003816. doi: 10.1371/journal.pgph.0003816 (PMC12077790; doi:10.1371/journal.pgph.0003816)
Supplement: S1 Table — (PDF) [file pgph.0003816.s001.pdf]

Supplementary Table 1: Formulae for β-cell function and insulin resistance markers

Markers	Definition/formula	Units	References
**β-cell function**			
Insulinogenic index	Change in insulin over change in glucose in first 30 minutes following OGTT.	(mU/L/mg/dL)	(27)
Oral disposition index	Insulinogenic index/1/fasting insulin	(mU/L,/ (mgdL) (mU/L)-1	(28)
HOMA-β	(20* Fasting blood insulin (FBI)/(Fasting plasma glucose (FPG)-3.5)	(mU/L, mmol/L)	(27)
**Insulin resistance**			
HOMA-IR	(FBI *FPG)/22.5	(mU/L, mmol/L)	(27)
Matsuda index	1000/√FPG*FBI) (MPG)*(MPI)	(mU/L, mg/dL)	(27)

AUC, area under the curve; HOMA-β, Homeostatic model assessment-β; HOMA-IR, HOMA-Insulin Resistance; OGTT, Oral glucose tolerance test; MPG, mean plasma glucose at 0, 30 and 120 minutes; MPI, mean of plasma insulin at 0, 30, and 120 minutes.
